# Macroevolutionary patterns of bumblebee body size: detecting the interplay between natural and sexual selection

**DOI:** 10.1002/ece3.65

**Published:** 2012-01

**Authors:** Raúl Cueva del Castillo, Daphne J Fairbairn

**Affiliations:** 1 Lab. de Ecología, UBIPRO, UNAM, FES IztacalaA. P. 314, México 54090, Edo. México, México; 2 Department of Biology, University of California at RiversideRiverside, California 92521

**Keywords:** Body size, bumblebees, natural selection, sexual dimorphism, sexual selection

## Abstract

Bumblebees and other eusocial bees offer a unique opportunity to analyze the evolution of body size differences between sexes. The workers, being sterile females, are not subject to selection for reproductive function and thus provide a natural control for parsing the effects of selection on reproductive function (i.e., sexual and fecundity selection) from other natural selection. Using a phylogenetic comparative approach, we explored the allometric relationships among queens, males, and workers in 70 species of bumblebees (*Bombus* sp.). We found hyperallometry in thorax width for males relative to workers, indicating greater evolutionary divergence of body size in males than in sterile females. This is consistent with the hypothesis that selection for reproductive function, most probably sexual selection, has caused divergence in male size among species. The slope for males on workers was significantly steeper than that for queens on workers and the latter did not depart from isometry, providing further evidence of greater evolutionary divergence in male size than female size, and no evidence that reproductive selection has accelerated divergence of females. We did not detect significant hyperallometry when male size was regressed directly on queen size and our results thus add the genus *Bombus* to the increasing list of clades that have female-larger sexual size dimorphism and do not conform to Rensch's rule when analyzed according to standard methodology. Nevertheless, by using worker size as a common control, we were able to demonstrate that bumblee species do show the evolutionary pattern underlying Rensch's rule, that being correlated evolution of body size in males and females, but with greater evolutionary divergence in males.

## Introduction

In sexual species, adult males and females often differ noticeably in characteristics other than primary sexual traits. A great many secondary sexual dimorphisms have been described, often as key identifying features of the species involved, but of these, differences in adult body size are the most pervasive and have been quantified and compared in numerous species of animals and plants (e.g., reviewed in Fairbairn 1997; Geber et al. 1999; Blanckenhorn 2005; Ruckstuhl and Neuhaus 2005; Fairbairn et al. 2007). Sexual size dimorphisms evolve because body size tends to be related to reproductive success through different pathways in females and males, often correlating most strongly with fecundity in females and with mating success in males. As result of these differences the body size that conveys maximal fitness (i.e., the optimal body size) often differs between the sexes. Sexual size dimorphism can also arise through other aspects of adaptation to sex-specific reproductive roles such as sex-specific foraging or dispersal strategies (i.e., reproductive niche dimorphism) or even as an adaptation to reduce intersexual trophic competition, although this is likely less common (e.g., see reviews in Hedrick and Temeles 1979; Reiss 1989; Shine 1989; Fairbairn 1997; Blanckenhorn 2005; Ruckstuhl and Neuhaus 2005; Fairbairn et al. 2007). The impact of sexual selection on sexual size dimorphism has been well established in many studies of individual species as well as in many phylogenetically controlled comparisons among species. Similarly, there is no doubt that large female size is favored by fecundity selection in taxa where females mature large numbers of eggs or live young within their abdomens, as in most fish, insects, and spiders (e.g., see references above and Roff 1991, 1992). However, the impacts of other forms of natural selection on sexual size dimorphism have been less thoroughly investigated and there is as yet no consensus about their relative importance. In this paper, we address this issue. We take advantage of the bumblebees’ social structure to separate out the effects of sexual selection, fecundity selection, and other forms of natural selection on the evolutionary divergence of body size in males and females.

In social insects individuals in the colony perform different functional roles. These different roles are associated with role-specific selective regimes that favor genetic integration of traits specific to each role and adaptive divergence role-specific phenotypes (Kovacs et al. 2010). In the social hymenopterans, females are divided into queen and worker castes (Wilson 1971). The queen is the only reproductive female in the colony, and the workers are sterile females that rear and protect the offspring and carry provisions to the nest. The sexes are similar in morphology but differ in size, with females generally larger than males (Stubblefield and Seger 1994; Gadagkar 1996). Males serve only to inseminate females and are both smaller and shorter lived than workers or queens (Stubblefield and Seger 1994). In bumblebees (*Bombus* sp.) queens tend to be larger than both males and workers and store large quantities of fat that are consumed during their hibernation (Richards 1946; Cumber 1949; Pereboom 2001). Aside from the differences in size and fat storage, the workers are identical to queens in external morphology.

As we might expect, in bees body size tends to be positively correlated with fecundity in females (Honěk 1993) and with mating success in males (Paxton 2005). However, in both sexes and both female castes body size also influences nonreproductive traits such as thermoregulation efficiency (Bishop and Armbruster 1999), mobility (Kapustjanskij et al. 2007), and competitive capacity associated with access to resources (Inoue and Yokoyama 2006; Inoue et al. 2007), and so is likely to be subject to selective pressures other than sexual and fecundity selection. Body size polymorphisms in contemporary social insect species are evidence of past selection within castes and sexes favoring different optimal body sizes. For example, the larger size of queens probably reflects selection for high fecundity and possibly also for high mating success (Kovacs et al. 2008) in addition to the various aspects of selection on nonreproductive traits. Similarly, male size must reflect selection for increased mating success in addition to selection on nonreproductive traits. Workers, as sterile females, experience neither sexual nor fecundity selection.

Bumblebees are often described as a primitive eusocial group as their social organization is simpler than that of other honeybees (Gadagkar 1996). In *Bombus*, except for a few tropical species, each colony contains only one queen (i.e., monogyny; Michener 1974). Perhaps, the main difference between bumblebees and other bees is that with a few exceptions they are annual organisms. Fertilized queens emerge from their hibernacula in late winter or early spring and establish new nests for a first generation of workers that will help them to set their colonies. During the first phase of the colony, a queen forages actively to gather nectar and pollen for nest provisioning. She moulds the pollen into a lump within which she lays her eggs. This lump is covered by a layer of wax mixed with pollen. She then incubates her brood by sitting on a groove on the top of the pollen lump. During this time the queen maintains close contact with her eggs and consumes her nectar reserves. Once the eggs have hatched, the queen has to forage to provide pollen to the offspring and replenish her nectar reserves. Once she has established a colony of sterile female workers, her main activity is to lay more eggs, while workers maintain the colony and forage for food (for details see Alford 1975). After producing the first generation of workers, the queen biases her offspring production in favor of new queens and males. The males depart from the colony soon after they have become adults to forage on flowers and search for mates. The young queens initially remain in their natal nest but unlike workers, they do not provide resources. Instead, they devote their time to foraging and increasing their own fat reserves until they too eventuality leave the nest to find mates during summer and fall (Goulson 2003).

Historically it has been considered that bumblebee females are monandrous (Duvoisin et al. 1999; Ayasse et al. 2001; Sauter et al. 2001; Colonello and Hartfelder 2005). However, some levels of polyandry have been reported in some species (Schmid–Hempel and Schmid–Hempel 2000; Paxton et al. 2001; Payne et al. 2003). After mating, the queens store sperm in their spermathecae until the following spring. After the hibernation period, the stored sperm are used to fertilize the eggs and found new colonies. If monandry is the rule in bumblebees and because in general they have highly male-biased populations (Bourke 1997), the opportunity for sexual selection on males must be strong (Baer 2003; Brown and Baer 2005). In some species males establish territories to get access to reproductive females, whereas in other species males are not territorial and actively seek out females (Williams 1991). In at least some species, larger males have the advantage in male–male competition (Alcock and Alcock 1983; Williams 1991; Paxton 2005). Nonetheless, many bumblebee species are protandrous (Bourke 1997; Beekman and Van Stratum 1998). Protandrous males actively look for virgin queens and may thus achieve a high mating success (Wiklund and Fagerström 1977; Bulmer 1983; Bourke 1997). However, there is a potential trade-off between protandry and body size. In hymenoptera, the degree sexual size dimorphism between queens and males is significantly correlated with the degree of sexual bimaturism (Blanckenhorn et al. 2007a). Thus, early maturation by males seems to come at the cost of smaller size, which may put these males at a disadvantage in direct male–male competition with large males (Wiklund and Fagerström 1977; Thornhill and Alcock 1983).

In this study, we analyze the evolutionary divergence in body size and sexual size dimorphism among *Bombus* species using a series of allometric predictions. We expect selection to act more strongly on queen and male traits than on worker-specific traits because workers, being sterile, experience selection only indirectly through their effects on colony success (Linksvayer and Wade 2009; Kovacs et al. 2010). Thus, a comparison of the evolutionary divergence of queens and males to that of workers should reveal the effects of selection on reproductive function (i.e., fecundity and sexual selection) within the context of largely shared patterns of ecological divergence.

Strong correlations between sexes are typical of most insect and vertebrate clades that have been examined for evolutionary allometries, including the hymenoptera (Fairbairn 1997; Blanckenhorn et al. 2007a,b; Fairbairn et al. 2007). These likely arise because of high genetic correlations between sexes (Poissant et al. 2010) in combination with species-specific adaptations to different ecological niches, for example, associated with foraging or dispersal strategies. In spite of these high correlations (typically > 0.9), the ratio of male to female body size often varies considerably among species within a given clade, indicating at least some independence of the evolutionary trajectories of body size in the two sexes. One might expect that genetic correlations between female castes would be as strong or stronger than those between sexes because the castes are genetically identical, differences being caused entirely by differential gene regulation during development. If so, the divergence of queens from workers may be more evolutionarily constrained than that between queens and males. However, contrary to this expectation, Kovacs et al. (2010) found only very low and nonsignificant correlations between queens and workers for any body size traits in the social wasp, *Vespula maculifrons.* Thus, caste dimorphism may actually evolve more readily than sexual dimorphism.

In many clades, the sexual size ratio changes systematically with mean size, either increasing or decreasing as body size increases (Rensch 1950; Fairbairn 1997; Fairbairn et al. 2007; Webb and Freckleton 2007). The former pattern is very common in taxa where males average larger than females, while the latter occurs in at least some clades in which females are the larger sex. Together these allometric trends are known as Rensch's rule. Both patterns are caused by greater evolutionary divergence in male size than in female size (i.e., greater variance among species for males than for females) combined with an underlying positive correlation between sexes. For many clades of both insects and vertebrates, this allometric trend can be attributed to sexual selection acting on male body size (e.g., for recent analyses and reviews, see Fairbairn 1997; Blanckenhorn et al. 2006; Fairbairn et al. 2007; Stillwell et al. 2010). The converse trend, where female size varies more than male size, is much less common but seems to be the rule in spiders, where it is posited to be caused by fecundity selection on females (Foellmer and Moya–Laraño 2007), as well as owls and some solitary bees (Stubblefield and Seger 1994; Abouheif and Fairbairn 1997; Fairbairn 1997; Blanckenhorn et al. 2007b; Webb and Freckleton 2007).

Based on these common allometric trends and assuming that patterns of selection differ among the bumblebee female castes (queens and workers) and males, we hypothesize the following allometric relationships (i.e., departures from isometry) for bumblebees:

1. *Allometry between queens and workers*: Because queens establish the colony in spring and must forage and tend the first generation of workers, they must be subject to much the same selection for foraging and brood care traits that workers are. Thus, differences between queens and workers in the degree of evolutionary divergence among species are likely to be due to selection on queens for reproductive function (mainly fecundity selection but possibly also sexual selection). If the evolutionary divergence in queen size has been driven at least in part by variation among species in the intensity of selection for reproductive function, we predict that the regression of queen size on worker size should have a slope greater than 1.
2. *Allometry between males and workers*: Males do not forage for or otherwise care for brood, and hence should not be subject to the same selection for these abilities as queens and workers. However, males are subject to sexual selection, which is clearly related to rapid divergence of male size in other animal clades. If the diversifying effect of sexual selection on males exceeds that of natural selection on workers, we predict that the regression of male size on worker size will have a slope greater than 1.
3. *Allometry between males and queens*: A slope greater than 1 will indicate that evolutionary divergence caused by sexual selection on males has exceeded that caused by reproductive selection on queens (fecundity selection and possibly sexual selection), and also selection for foraging and brood care. This is the trend that we expect, based on Rensch's rule and previous allometric studies of Hymenoptera (Blanckenhorn et al. 2007a,b), but it has not previously been determined for bumblebees.

## Methods

### Morphological data

A search of *Bombus* specimens was performed in the Museums of Entomology of the University of California at Riverside and Berkeley, and the *Bombus* collections of the Natural History Museum of Los Angeles (NHM-LA), and the California Academy of Sciences (CAS). We then looked for species represented in the phylogeny of 218 bumblebee species derived by Cameron et al. (2007) and based on DNA sequence data. To include a species in the study, we set a minimum sample size of two individuals of each sex and caste (queens, workers, and males). However, if it was possible, we measured five individuals in each category. Moreover, because there is a high variation in body size due to environmental conditions and geographic distribution (see Alford 1975; Goulson 2003), we took care to sample from the full range of available sizes for each category by taking one individual from each extreme of the phenotypic distribution and the rest at random. We measured head width (maximal distance between the distal surfaces of the eyes measured in dorsal aspect) and the thorax width (intertegular distance). Measurements were taken using ocular micrometers with a precision to the nearest 0.082 mm on Leica MZ 75 (Leica Microsystems Wetzlar; Germany) and Zeiss SV6 (Carl Zeiss MicroImaging, Thornwood, NY) microscopes (8´ magnifications). In addition to the measurements we obtained for 65 species in the collections, we included measurements for another five species (*B. beaticola*, *B. hypocrita, B. ignitus, B. pseudobaicalensis*, and *B. schrencki*) from published sources (Inoue and Yokoyama 2006; Inoue et al. 2008; see Table 1).

**Table 1:** Mean values for thorax and head width of males, queens, and workers and Sexual Dimorphism Index; SDI (Lovich and Gibbons 1992) for 70 colonial *Bombus* species. Sample sizes are shown in parentheses

										SDI: (Female/Male)-1			
										
	Males			Queens			Workers			Queen/Male		Worker/Male	
								
Species	Thorax width	Head width		Thorax width	Head width		Thorax width	Head width		Thorax	Head	Thorax	Head
*B. affinis*	3.80	3.07	(3)	5.54	3.92	(5)	3.52	2.74	(5)	0.46	0.28	−0.07	−0.11
*B. appositus*	3.58	2.70	(5)	5.18	3.52	(5)	3.86	2.72	(5)	0.45	0.30	0.08	0.01
*B. ardens*	3.76	3.28	(4)	5.30	3.56	(4)	3.79	2.86	(5)	0.41	0.09	0.01	−0.13
*B. atratus*	3.53	3.24	(5)	5.66	3.75	(4)	3.92	2.89	(5)	0.60	0.16	0.11	−0.11
*B. atripes*	4.90	3.45	(2)	6.24	4.42	(5)	4.66	3.46	(5)	0.27	0.28	−0.05	0.00
*B. auricomus*	5.70	3.95	(4	6.56	4.22	(5)	4.76	3.34	(5)	0.15	0.07	−0.16	−0.15
*B. balteatus*	3.42	2.44	(5)	5.76	3.66	(5)	3.70	2.68	(5)	0.68	0.50	0.08	0.10
*B. beaticola*	4.20	3.50	*	5.40	4.00	*	4.00	3.30	*	0.29	0.14	−0.05	−0.06
*B. bifarius*	2.82	2.36	(5)	4.36	2.92	(5)	2.94	2.30	(5)	0.55	0.24	0.04	−0.03
*B. bimaculatus*	3.40	2.58	(5)	5.04	3.48	(5)	3.38	2.70	(5)	0.48	0.35	−0.01	0.05
*B. borealis*	4.20	2.75	(2)	4.98	3.36	(5)	3.96	2.70	(5)	0.19	0.22	−0.06	−0.02
*B. californicus*	3.54	2.76	(5)	5.52	3.50	(5)	3.98	2.86	(5)	0.56	0.27	0.12	0.04
*B. centralis*	3.04	2.42	(5)	4.48	3.00	(5)	3.06	2.40	(5)	0.47	0.24	0.01	−0.01
*B. crotchii*	4.72	3.60	(5)	6.44	4.06	(5)	4.36	3.16	(5)	0.36	0.13	−0.08	−0.12
*B. dahlbomii*	4.36	3.30	(5)	6.78	4.44	(5)	3.78	2.74	(5)	0.56	0.35	−0.13	−0.17
*B. diligens*	3.83	2.97	(3)	5.62	3.68	(5)	3.76	2.84	(5)	0.47	0.24	−0.02	−0.04
*B. diversus*	4.73	3.64	(5)	4.59	3.32	(2)	3.85	3.03	(4)	−0.03	−0.09	−0.19	−0.17
*B. ephippiatus*	3.38	2.76	(5)	5.08	3.40	(5)	3.64	2.74	(5)	0.50	0.23	0.08	−0.01
*B. fervidus*	3.75	2.90	(2)	4.64	3.38	(5)	3.80	2.88	(4)	0.24	0.17	0.01	−0.01
*B. fraternus*	3.14	2.44	(5)	4.10	2.80	(5)	3.08	2.30	(5)	0.31	0.15	−0.02	−0.06
*B. frigidus*	3.14	2.46	(4)	3.62	2.70	(4)	3.00	2.25	(3)	0.15	0.10	−0.04	−0.09
*B. funebris*	5.20	3.85	(5)	6.28	4.42	(5)	4.08	3.10	(4)	0.21	0.15	−0.22	−0.19
*B. griseocollis*	4.60	3.14	(5)	5.83	3.73	(5)	4.08	2.80	(5)	0.27	0.19	−0.11	−0.11
*B. honshuensis*	4.42	3.10	(5)	5.46	3.56	(5)	3.70	2.66	(5)	0.24	0.15	−0.16	−0.14
*B. hortorum*	3.50	2.60	(2)	4.77	3.10	(5)	3.52	2.64	(5)	0.36	0.19	0.01	0.02
*B. huntii*	3.13	2.43	(5)	4.66	3.30	(5)	3.22	2.44	(5)	0.49	0.36	0.03	0.00
*B. hyperboreus*	4.08	2.90	(4)	6.04	3.56	(5)	5.40	3.67	(3)	0.48	0.23	0.32	0.27
*B. hypnorum*	3.53	2.63	(3)	5.14	3.30	(5)	3.34	2.40	(5)	0.46	0.25	−0.05	−0.09
*B. hypocrita*	5.80	4.50	*	7.70	7.10	*	5.40	4.20	*	0.33	0.58	−0.07	−0.07
*B. ignitus*	6.40	4.90	*	8.10	5.80	*	6.30	4.80	*	0.27	0.18	−0.02	−0.02
*B. impatiens*	3.42	2.59	(5)	5.21	3.69	(5)	3.38	2.67	(5)	0.52	0.42	−0.01	0.03
*B. jonellus*	2.92	2.22	(5)	4.76	3.08	(5)	3.16	2.34	(5)	0.63	0.39	0.08	0.05
*B. lapidarium*	3.00	2.60	(2)	5.20	3.48	(5)	3.18	2.40	(5)	0.73	0.34	0.06	−0.08
*B. lapponicus*	3.03	2.47	(4)	3.90	3.60	(5)	2.90	2.45	(2)	0.29	0.46	−0.04	−0.01
*B. lucorum*	4.16	2.96	(5)	4.40	3.13	(5)	3.75	2.83	(4)	0.06	0.06	−0.10	−0.04
*B. medius*	3.24	2.59	(4)	5.66	3.94	(5)	3.64	2.82	(5)	0.75	0.52	0.12	0.09
*B. melanopygus*	3.00	2.46	(5)	4.82	3.34	(5)	3.14	2.44	(5)	0.61	0.36	0.05	−0.01
*B. mesomelas*	3.35	2.50	(2)	4.05	2.85	(2)	3.20	2.36	(5)	0.21	0.14	−0.04	−0.06
*B. mexicanus*	3.15	2.60	(2)	6.04	3.76	(5)	3.64	2.72	(5)	0.92	0.45	0.16	0.05
*B. mixtus*	2.96	2.26	(5)	4.24	3.04	(5)	3.10	2.56	(5)	0.43	0.35	0.05	0.13
*B. morio*	3.65	2.60	(2)	4.66	3.28	(5)	3.48	2.52	(5)	0.28	0.26	−0.05	−0.03
*B. morrisoni*	4.76	3.30	(5)	6.48	4.11	(5)	4.14	2.99	(5)	0.36	0.25	−0.13	−0.09
*B. nevadensis*	5.18	3.58	(5)	6.08	3.78	(5)	5.10	3.10	(5)	0.17	0.06	−0.02	−0.13
*B. occidentalis*	3.66	2.86	(5)	5.42	3.50	(5)	3.42	2.54	(5)	0.48	0.22	−0.07	−0.11
*B. pennsylvanicus*	4.28	3.80	(5)	5.44	3.82	(5)	3.96	3.06	(5)	0.27	0.01	−0.07	−0.19
*B. perplexus*	3.10	2.76	(5)	4.98	3.43	(5)	3.18	2.46	(5)	0.61	0.24	0.03	−0.11
*B. polaris*	2.70	2.15	(2)	5.03	3.17	(3)	4.08	2.56	(5)	0.86	0.47	0.51	0.19
*B. pratorum*	3.04	2.54	(5)	4.36	2.98	(5)	3.10	2.44	(5)	0.43	0.17	0.02	−0.04
*B. pseudobaicalensis*	4.80	3.80	**	6.50	4.70	**	4.60	3.70	**	0.35	0.24	−0.04	−0.03
*B. pullatus*	4.03	3.15	(5)	5.90	4.05	(4)	4.50	3.16	(5)	0.46	0.29	0.12	0.00
*B. rubicundus*	4.41	3.10	(5)	6.11	3.96	(5)	3.98	2.74	(5)	0.39	0.28	−0.10	−0.12
*B. rufofasciatus*	3.64	2.60	(5)	4.60	3.09	(5)	3.13	2.26	(5)	0.26	0.19	−0.14	−0.13
*B. schrencki*	4.50	2.60	**	6.50	4.60	**	4.30	3.60	**	0.44	0.77	−0.04	0.38
*B. sichelii*	3.86	2.78	(5)	5.05	3.48	(4)	3.16	2.26	(5)	0.31	0.25	−0.18	−0.19
*B. sitkensis*	3.16	2.48	(5)	4.33	3.20	(4)	3.16	2.44	(5)	0.37	0.29	0.00	−0.02
*B. sonorus*	4.16	3.10	(5)	6.08	4.00	(4)	3.94	2.78	(5)	0.46	0.29	−0.05	−0.10
*B. steindachneri*	3.96	2.94	(5)	5.67	3.82	(5)	3.70	2.95	(5)	0.43	0.30	−0.07	0.00
*B. subterraneus*	3.72	2.98	(5)	5.65	3.68	(4)	3.80	2.60	(2)	0.52	0.23	0.02	−0.13
*B. sylvarum*	3.33	2.55	(4)	4.52	3.02	(5)	3.18	2.48	(2)	0.36	0.18	−0.05	−0.03
*B. sylvicola*	3.10	2.50	(5)	4.14	2.92	(5)	3.18	2.24	(5)	0.34	0.17	0.03	−0.10
*B. ternarius*	3.06	2.54	(5)	4.70	3.30	(5)	3.10	2.44	(5)	0.54	0.30	0.01	−0.04
*B. terrestris*	4.23	3.90	(3)	5.69	3.83	(5)	3.61	2.79	(5)	0.35	−0.02	−0.15	−0.28
*B. terricola*	3.74	2.88	(5)	4.81	3.44	(5)	3.42	2.60	(5)	0.29	0.19	−0.09	−0.10
*B. vagans*	2.94	2.44	(5)	4.42	3.16	(5)	3.22	2.54	(5)	0.50	0.30	0.10	0.04
*B. vandykei*	2.96	2.50	(5)	3.92	3.12	(5)	3.00	2.44	(5)	0.32	0.25	0.01	−0.02
*B. veteranus*	3.18	2.55	(4)	4.96	3.22	(5)	3.24	2.42	(5)	0.56	0.26	0.02	−0.05
*B. volucelloides*	4.03	3.03	(4)	6.26	3.98	(5)	4.04	2.96	(5)	0.55	0.31	0.00	−0.02
*B. vosnesenskii*	2.98	2.56	(5)	5.30	3.67	(5)	3.41	2.51	(5)	0.78	0.43	0.14	−0.02
*B. weisi*	3.48	2.73	(4)	4.40	3.18	(5)	3.10	2.52	(5)	0.26	0.16	−0.11	−0.08
*B. wurflenii*	3.94	2.86	(5)	5.40	3.65	(2)	3.70	2.75	(2)	0.37	0.28	−0.06	−0.04
Mean	3.79	2.91		5.29	3.63		3.71	2.77		0.41	0.25	−0.01	−0.04
STD	0.77	0.53		0.88	0.67		0.65	0.46		0.18	0.14	0.11	0.10
CV	20.4	18.23		16.6	18.38		17.39	16.41					

All the units are expressed in mm.

* = Inoue and Yokoyama 2006

** = Inoue et al. 2008.

The overall mean, standard deviation (STD) are shown for female castes, males, and SDI. In addition, coefficients of variation (CV) for the morphological variables are provided.

### Phylogeny and comparative analyses

**Figure 1:**
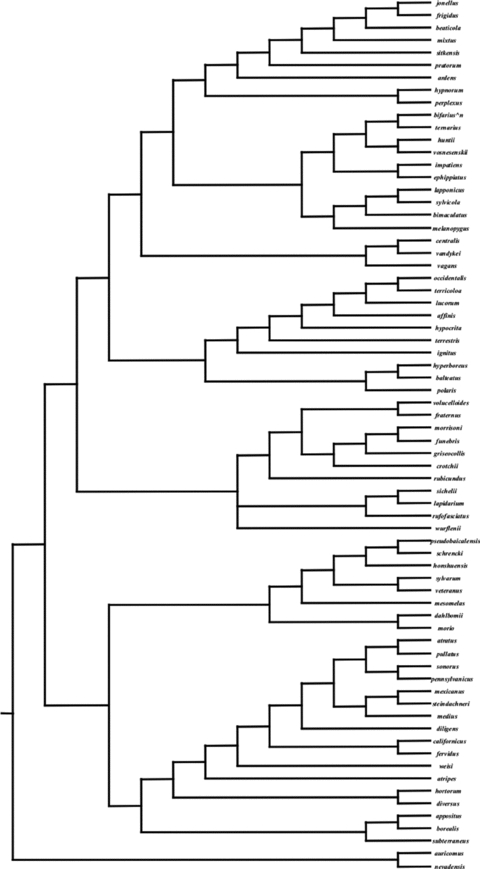
Phylogenetic relationships for 70 bumblebee species (Table 1) considered on the comparative analyses, adapted from Cameron et al. (2007).

We used the bumblebee phylogeny from Cameron et al. (2007) to determine the relationships among the 70 species that we included in our comparative analysis for the allometric regression. The phylogenetic tree for these species was constructed using Mesquite Software version 1.07 (see Maddison and Maddison 2004). Tree branch length in all cases was equal (length = 1.0; see Pagel 1992), except in polytomies. Because COMPARE (see below) does not accept polytomies (Martins 2004), for these cases we assigned a branch length of 0.001 (Fig. 1). Previous to comparative analyses, the morphometric variables were log transformed. Using the independent contrast module of COMPARE 4.6b (Martins 2004), we obtained the Felsenstein independent contrasts for the thorax and head width of the three bumblebee castes.

The allometric relationships between the bumblebee castes were characterized using model II regression also known as major axis regression (Sokal and Rohlf 1995), considering as *h*_0_:β = 1 (isometry). Model II techniques provide a more appropriate estimate of the line of allometry than model I (Ordinary Least Squares; OLS) regression, because residual variance is minimized in both *x* and *y* dimensions, rather than the *y* dimension only (Sokal and Rohlf 1995; Warton et al. 2006). Model II regressions were performed in (S)MATR (Falster et al. 2006), which provides for the equivalent of analysis of covariance. The program first fits slopes within each group, with confidence intervals calculated following Pitman (1939), then tests for statistical differences in slopes between groups, using methods outlined by Warton and Weber (2002).

Because the phylogeny (Fig. 1) includes a polytomy, we subtracted one additional degree of freedom from each regressed model (see Garland and Diaz–Uriarte 1999). We used one-tailed probabilities for hypothesis testing because each of our a priori hypothesis is directional.

## Results

As expected, we found that queens were the largest and workers the smallest of the three bumblebee morphs, and all three morphs show considerable variation among the 70 species in our sample for both head and thorax width (Table 1). In queens, mean thorax width ranges from 3.62 to 8.10 mm and head width from 2.70 mm to 7.10 mm, a 2.2-fold difference in thorax size and 2.6-fold in head width. Males and workers show similar ranges (thorax width: 2.70–6.40 mm for males and 2.90–6.30 mm for workers; head width 2.15–4.90 for males and 2.24–4.80 for workers), with a 2.1- to 2.3-fold difference in linear size between the smallest and the largest species. Since all *Bombus* species are presumed to have evolved from a single common ancestor, these ranges denote considerable divergence in the size of all three morphs during the evolution of the clade.

**Figure 2:**
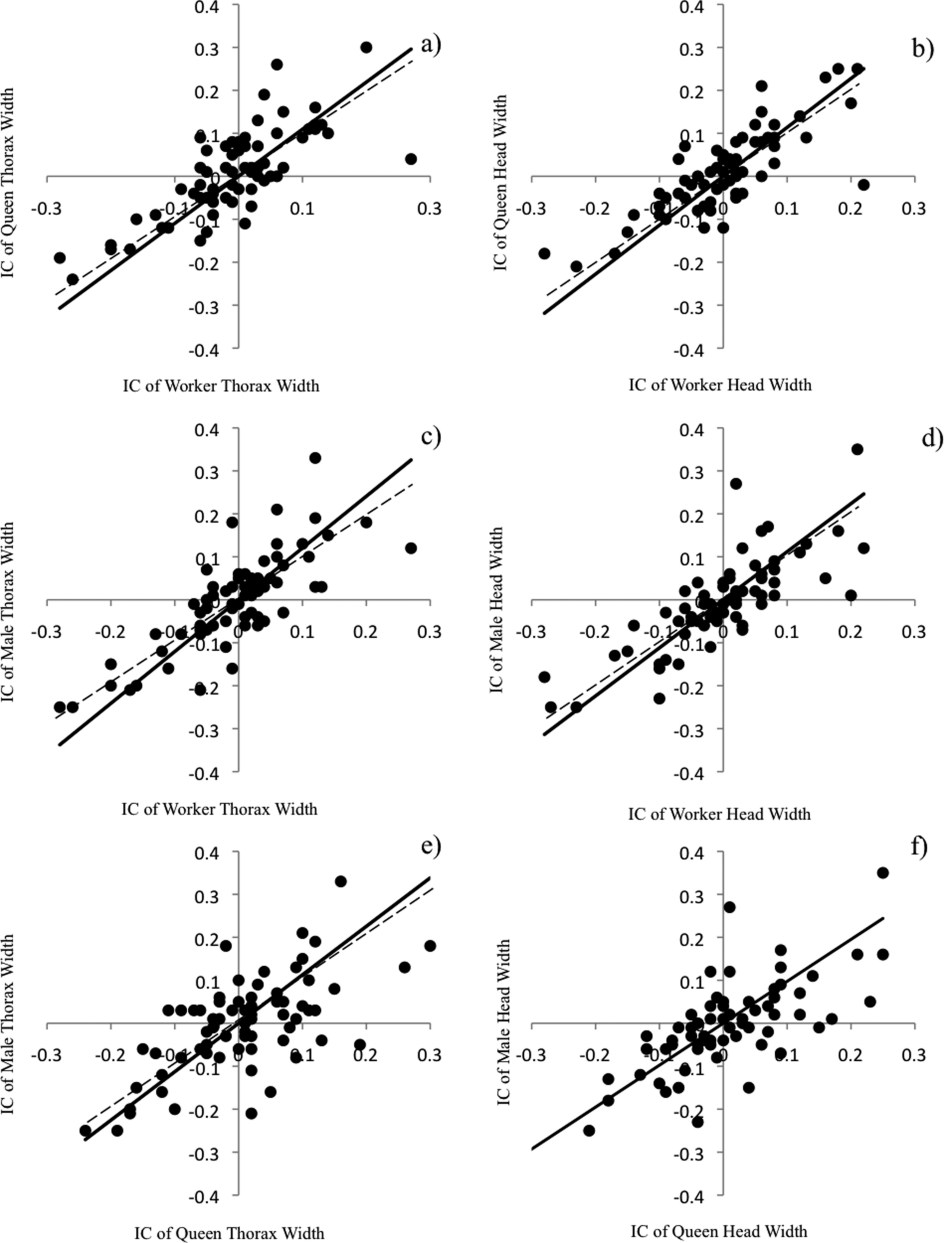
Allometric major axis regressions of independent contrasts (IC) for *Bombus* sp. males, workers, and queens. Thorax width: panels (A), (C), (E). Head width: panels (B), (D), (F). Dashed lines indicate isometry (β = 1).

The results of the independent contrasts analysis indicate strong coevolution of the queens, males, and workers (Table 2). Although the slopes of the regressions of the sexual adults on workers are all greater than 1.0 (Table 2; Fig. 2A–D), only the relationship between male thorax width and worker thorax width differs significantly from isometry (i.e., has a slope > 1.0). Thus, with the exception of thorax width in males, it seems that evolutionary divergence has been similar in the sexual and sterile castes.

**Table 2:** Results of model II allometric regressions of the independent contrasts of thorax and head width of queens, workers, and males bumblebees considered in the comparative study

Regressed variables	Model II slope	UCI	LCI	*r*^2^	*F*	*P_regr_*	*r*	*P_corr_*
Queen on worker thorax width	1.096	1.350	0.893	0.586	0.801	0.187	0.766	<0.0001
Queen on worker head width	1.138	1.354	0.960	0.670	2.294	0.067	0.819	<0.0001
Male on worker thorax width	1.205	1.457	1.002	0.631	4.022	0.024	0.794	<0.0001
Male on worker head width	1.119	1.358	0.926	0.621	1.410	0.119	0.788	<0.0001
Male on queen thorax width	1.128	1.524	0.844	0.415	0.697	0.203	0.644	<0.0001
Male on queen head width	0.976	1.248	0.762	0.501	0.039	0.423	0.708	<0.0001

The upper (UCI) and lower (LCI) confidence intervals (95%) of the model II, explained variance (*r*^2^), *F*, and *P* values are shown. In all cases, df = 1, 67. *P_regr_* refers to the null hypothesis of isometry (*h*_0_:β = 1). Also shown are the Pearson coefficients of correlation, *r*, and their associated probabilities (*P_corr_*).

The hyperallometry between males and workers for thorax width is consistent with a priori hypothesis 2 that male thorax width has diverged in response to sexual selection. The slope for males is also significantly larger than that for females (χ^2^ = 4.27; df = 1, *P* = 0.039), indicating that male thorax width has diverged more than queen thorax width during the evolution of the clade. This trend should produce hyperallometry between males and queens for thorax width (i.e., allometry consistent with Rensch's rule), but the regression of males on queens fails to pick this up: the allometric slope is not significantly greater than 1.0 (Fig. 2E).

## Discussion

In spite of abundant variation among species in both thorax width and head width, we found little evidence that the rate of divergence from the ancestral size has differed between queens and workers. Our phylogenetically controlled regressions did not detect significant departures from isometry for head or thorax width. Thus, we found no support for hypothesis 1. Selection for reproductive function does not seem to have caused increased evolutionary change in queen size relative to worker size. We did find significant hyperallometry for thorax width when males were regressed on workers, which supports our hypothesis 2, that sexual selection has caused increased evolutionary divergence in males relative to workers. The slope of the regression of males on workers was also significantly steeper than that for females on workers, which suggests a significantly greater evolutionary divergence of males than of queens, when controlled for the variation in worker size. These results would lead one to expect significant hyperallometry when male thorax size is regressed on queen thorax size, but although the slope was greater than 1.0, it was not significantly so. Thus, although sexual selection on male size is often associated with between-sex allometry consistent with Rensch's rule (Fairbairn 1997; Fairbairn et al. 2007; Webb and Freckleton 2007), we found no evidence of hyperallometry when males were regressed on queens. *Bombus* thus joins the increasing list of clades in which reproductive females are larger than males and Rensch's rule does not hold (Abouheif and Fairbairn 1997; Fairbairn 1997; Blanckenhorn et al. 2007b; Foellmer and Laraño 2007; Webb and Freckleton 2007).

Both size polymorphisms and patterns evolutionary scaling of body size arise from the interplay between sexual and natural selection acting on body size (Slatkin 1984; Hedrick and Temeles 1989; Shine 1989; Fairbairn 1997; Fairbairn et al. 2007). In bumblebees, body size is likely to have a strong influence on the fitness of all three morphs (queens, males, and workers) and the consistent differences in average size indicate that the size that maximizes fitness (the optimal body size) must be morph specific. (Strictly speaking, workers have no direct fitness because they do not reproduce, but their performance influences the colony fitness. Selection should favor the worker body size that maximizes colony fitness.) In general larger bumblebees have larger foraging ranges (Pyke 1978; Kapustjanskij et al. 2007) and can be more efficient finding flowers and collecting nectar (Pyke 1978; Macuda et al. 2001). Since all three bumblebee morphs (queens, males, and workers) forage during at least some stages of their lives, one would predict that selection for foraging efficiency would influence the optimal size in all three morphs. Large size is likely to confer additional benefits for bumblebee queens because of the relationships between size and success in competition for resources, fecundity, thermoregulation, and parental care (Owen 1988). At the beginning of the spring solitary and inseminated queens start to look for places to establish their nests. During this critical phase of their life cycle their probability of failure is high. The young queens fight for their potential nesting places, and intra- and interspecific nest usurpation attempts are common. In these contests large queens probably have the advantage in both defending and usurping nests (Plowright and Laverty 1984). The fecundity of bees, as in other insects, is positively related with their body size (Honěk 1993), but also depends on their fat and water reserves (Alford 1969; Holm 1972). While these selective pressures probably account for the larger average size of queens when compared to both males and workers, they have not resulted in significant hyperallometry of size in queens relative to workers or males. Thus, although the optimal size is larger for queens than for males or workers, the net intensity of selection on queen body size has not varied sufficiently among species to cause more interspecific divergence in body size in queens than in the other morphs during the evolution of the clade.

Males also reap additional benefits from larger size because competition among males for mates favors larger males (Boomsma et al. 2005; Paxton 2005). Sexual selection on male bumblebees is likely to be intense because of the low levels of polyandry (Schmid–Hempel and Schmid–Hempel 2000; Paxton et al. 2001; Payne et al. 2003) and highly male-biased operational sex ratios typical of wild populations (Bourke 1997). The hyperallometry of male thorax size relative to worker size likely reflects the greater evolutionary divergence of males in response to this strong sexual selection. However, we did not detect significant hyperallometry when comparing males and queens: the slope of the regression of males on queens did not differ significantly from unity. This result seems at odds with our finding of a significantly higher slope for males than for females when regressed on workers. The apparent isometric scaling of males on queens is associated with the relatively low correlation between the sexes (*r* = 0.64) when compared to the correlations between males and workers (*r* = 0.79) or queens and workers (*r* = 0.77), which results in a very wide confidence interval for the major axis slope when males are regressed on queens. Thus, although male thorax size does seem to have diverged more than female thorax size during the evolution of the *Bombus* clade, Rensch's rule fails because of the relatively weak covariance between reproductive males and females.

Many arthropods and vertebrates in which females are the larger sex do not follow Rensch's rule (Abouheif and Fairbairn 1997; Fairbairn 1997; Blanckenhorn et al. 2007b; Foellmer and Moya–Laraño 2007; Webb and Freckleton 2007). Some, such as owls, solitary bees, and spiders, show the opposite pattern of allometry (i.e., a slope significantly less than 1.0 when male size is regressed on female size), which indicates that female size has diverged more than male size over the evolutionary history of the clade. However, most exceptions to Rensch's rule are simply cases where the regression slope does not differ significantly from 1.0, as we have found for bumblebees. By regressing males and queens on workers, we were able to detect hyperallometry of body size in males in spite the apparent isometry in the standard regression of males on the reproductive females; the queens. These results suggest that absence of allometry consistent with Rensch's rule should not be taken as evidence that sexual selection has not played a major role in body size evolution in a clade. In clades with female-larger size dimorphism, the evolutionary divergence of male body size caused by sexual selection may simply be matched or exceeded by strong diversifying selection on female size. Thus, as when interpreting patterns of static allometry, we must be cautious when inferring patterns of selection from patterns of evolutionary allometry or, conversely, when predicting allometry from patterns of sexual selection (Bonduriansky and Day 2003; Bonduriansky 2007; Bertin and Fairbairn 2007).
